# Concurrent radiochemotherapy in advanced hypopharyngeal cancer

**DOI:** 10.1186/1748-717X-5-39

**Published:** 2010-05-18

**Authors:** Valentina Krstevska, Igor Stojkovski, Dusko Lukarski

**Affiliations:** 1 Department of Head and Neck Cancer, University Clinic of Radiotherapy and Oncology, Skopje, Macedonia; 2 Department of Radiation Physics, University Clinic of Radiotherapy and Oncology, Skopje, Macedonia

## Abstract

**Background:**

Concurrent platinum-based radiochemotherapy has been recommended as a standard of care in patients with locally advanced squamous cell head and neck carcinomas. Unfortunately, there is a lack of level one evidence on best treatment approach for advanced hypopharyngeal cancer. This report aims to summarize the results of our study on concurrent radiochemotherapy in patients with advanced hypopharyngeal cancer.

**Methods:**

A retrospective analysis of 41 patients with stage III-IV hypopharyngeal cancer was performed. All patients were treated with three dimensional conformal radiotherapy and received 70 Gy in 35 fractions (2 Gy per fraction, 5 fractions per week). In dependence of the period when radiotherapy was realized, two different treatment techniques were used. Concurrent chemotherapy consisted of cisplatin 30 mg/m^2 ^given on a weekly basis.

**Results:**

The median age was 52 years (range 29-70). Stage IV disease was recognized in 73.2% of the patients. Complete response rates at the primary site and at the metastatic neck lymph nodes were 68.3% and 36.6%, respectively. A complete composite response was present in 27 patients (65.9%). Median follow-up was 13 months (range 7-36). Distant metastases as initial failure occurred in 7 patients (46.7%). The 2-year local relapse-free survival and regional relapse-free survival rates were 55.2% and 75.8%, respectively. The 2-year locoregional relapse-free survival rate was 51.3%. The 2-year disease-free survival and overall survival rates were 29.3% and 32.8%, respectively. Confluent mucositis was developed in 46.3% of patients. Leucopenia grade 1 was the most frequent hematological toxicity. The median weight loss at the end of treatment was 12% (range 5-21). The worst grade of late toxicity was most commonly pronounced in the skin and in the subcutaneous tissue.

**Conclusions:**

Based on unsatisfactory results in our study we suggest that the use of sequential radiochemotherapy or chemotherapy given concomitantly with altered fractionation radiotherapy with the implementation of intensity-modulated radiotherapy as radiotherapy technique could represent treatment approaches able to improve outcome in patients with advanced hypopharyngeal cancer.

## Background

Hypopharyngeal cancer is a rare disease representing about 0.5% of all human malignancies with an incidence of less than 1 per 100 000 population and constituting only 3-5% of all head and neck cancers [1,3]. Hypopharyngeal cancers are often at an advanced stage at diagnosis and are associated with a poor prognosis [4,6]. The reasons for the unfavourable prognosis of hypopharyngeal cancers are the strong tendency for extensive submucosal spread, the early occurrence of regional lymphatic involvement, and the relatively high rate of distant spread [7,8].

In the 1970s and 1980s, surgery, followed by postoperative radiotherapy was the standard form of therapy for advanced stage disease [9,10]. This radical approach of treatment, lead to the loss of natural speech function and impairment of swallowing ability with a consequent negative impact on the quality of life, and low cure rates, reported 5-year survival between 20.0% and 50.0% [1,2,7,11,12].

The necessity for improvement of survival rates and preserving organ function resulted in introduction of chemotherapy as a third treatment modality for patients with advanced hypoharyngeal cancer. The combined modality treatment was subject of analysis in two randomized trials. In the study of Beauvillain et al. [13] comparing chemotherapy plus radiotherapy with chemotherapy plus surgery plus radiotherapy, the outcome was better in the surgical arm. In the randomized trial conducted by the European Organization for Research and Treatment of Cancer (EORTC) Head and Neck Cancer Cooperative group, comparing chemotherapy plus radiotherapy with surgery plus radiotherapy, surgical and non-surgical groups had similar 5-year survival [14]. Both studies did not succeed to give evidence to support radical surgery for curative treatment for advanced hypoharynegal cancer. Also, the EORTC 24891 trial [15] demonstrating that laryngeal preservation by induction chemotherapy followed by definitive radiotherapy was a safe treatment alternative for patients with T2-T4 tumours, worked as a good basis for further investigations of non-surgical management of hypopharyngeal cancers [16].

Concurrent radiochemotherapy (CRCT) as definitive treatment for advanced head and neck including cancers arising from the hypopharynx has been studied in the past 15 years [17,23].

However, due to the low incidence, hypopharyngeal cancers grouped with other head and neck cancers usually represented only smaller subgroups with details of their treatment being rarely specifically reported [8,16]. The rarity of this disease, and the time needed for data collection could be accepted as an explanation for the absence of multicenter randomized clinical trials undertaken to evaluate the role of CRCT in the treatment of advanced hypopharyngeal cancer. Despite the fact that concurrent platinum-based radiochemotherapy was adopted as a standard of care in patients with locally advanced HNSCC [24], there is no level one evidence on best treatment [16], or agreement on treatment for advanced hypoharyngeal cancer [25].

In order to evaluate the results of non-surgical combined treatment approach we retrospectively analyzed patients with advanced hypopharyngeal cancer treated with chemotherapy consisting of cisplatin given on a weekly basis administered concurrently with external-beam radiotherapy performed using three dimensional conformal technique.

## Methods

Forty-one consecutive patients with newly diagnosed advanced stage III-IV squamous cell carcinoma of the hypopharynx treated with definitive CRCT, from January 2006 to October 2009 at the University Clinic of Radiotherapy and Oncology in Skopje were analyzed. Pre-treatment evaluations included history, physical examination, panendoscopy and biopsy, computed tomography (CT) and/or magnetic resonance imaging (MRI) of the hypopharyngeal and cervical region, chest x-ray, liver ultrasound and routine laboratory studies. Patients were staged according to the 2002 criteria of the American Joint Committee on Cancer [26]. Written informed consent was obtained from the patients for including in the study. A copy of the consent is available for review by the Editor-in-Chief of this journal.

### Radiotherapy

The patients were immobilized in supine position with a thermoplastic head and neck mask. They were treated by photons with beam qualities of 6 MV and 15 MV and electrons with energies 9-16 MeV. For the treatment planning, we used the Eclipse Version 7.3.10, a commercial 3D treatment planning system manufactured by Varian Medical Systems. The CT scanning was made for each patient in the treatment position with slice thickness of 0.5 cm.

The gross tumour volume of the primary tumour (GTVt70) and the metastatic lymph nodes (GTVn70) were defined as any visible tumour and the gross nodal disease revealed on imaging studies and/or physical examination. Neck lymph nodes were considered metastatic when their smallest axis diameter was greater then 1.0 cm. The clinical target volume (CTVt50) encompassed the GTVt70 plus a margin of 1.0-2.0 cm for the potential microscopic extension of the disease. In patients with negative neck lymph nodes the CTVn50 included the nodal regions in the neck at levels II-IV. In patients with clinically involved neck lymph nodes, CTVn50 included GTVn70 with a margin of 0.5-1.0 cm and also encompassed retropharyngeal lymph nodes and nodal regions at levels I-V. Level VI was included in CTVn50 only in cases when primary tumour invaded oesophagus. CTV50 was created by integration of CTVt50 and CTVn50. The planning target volumes were PTV50 and PTV70. The PTV50 provided a margin of 0.5 cm around CTV50. If there were no positive lymph nodes in the neck, the PTV70 encompassed the GTVt70 plus a 0.5 cm margin. In patients with nodal disease, the GTV70 was union of GTVt70 and GTVn70, and by adding a margin of 0.5 cm around it, we obtained PTV70.

The patients were treated by two different treatment techniques.

The first treatment technique used from January 2005 until January 2008, consisted of three stages. In the first stage we used semi-fields, where the upper neck was irradiated by two opposing lateral semi-fields, and the lower neck was irradiated by anterior and posterior semi-fields. For the posterior semi-field we used 15 MV photons, and for the other fields, 6 MV photons. This stage consisted of 23 fractions, 2 Gy each. In the second stage, comprising of 2 fractions, 2 Gy each, the lateral fields were reduced from the dorsal side in order to exclude the spinal cord from the fields. The dose to the shielded dorsal part of the PTV50 was delivered by two lateral electron fields, which were matched to the photon fields. In the third stage of the treatment, depending on the position and the volume of the PTV70, we used arrangements with 2 to 4 photon fields with beam quality 6 MV in lateral or oblique directions with occasional use of electron fields, delivering the remaining 20 Gy in 10 fractions. In this stage the spinal cord was completely out of field.

The second technique, started from February 2008, which we have named "oblique photon fields" technique, consisted of two stages. The idea was to eliminate the use of electron fields, because of the inconveniences that occur when matching photon and electron fields (the cold spots at the surface or the hot spots at greater depth). In the first stage we delivered 50 Gy in 25 fractions by 4 oblique isocentric photon fields of beam quality 6 MV. Two of the fields, the anterior ones, were positioned at gantry angles 300° and 60° and covered the whole PTV50. The posterior oblique fields were at gantry angles between 210° and 220° from the right side of the patient, and between 135° and 145° from the left side. The spinal cord was shielded in these fields, so they covered only part of the PTV50. The weight of the posterior fields was approximately 4 times smaller than the weight of the anterior ones. The second stage was identical to the third stage of the first technique.

### Chemotherapy

Chemotherapy consisted of cisplatin 30 mg/m^2 ^along with standard hydration and antiemetic prophylaxis given to the patients concomitantly with radiation on a weekly basis. Patients commenced chemotherapy on the same day as commencing radiotherapy. The full blood count and biochemistry were checked weekly before chemotherapy. The criteria of skipping or stopping chemotherapy were leucopoenia or experienced severe mucositis.

### Response assessment

Response evaluation was performed three months after completion of radiochemotherapy by physical examination, computed tomography CT and/or MRI, and endoscopy under general anaesthesia. For the primary tumour, a complete response was defined as complete disappearance of clinical and radiological evidence of disease with a complete recovery of larynx mobility. Partial response was defined as a regression of a 50% or more of the tumour volume and at least a partial recovery of the larynx mobility. A complete nodal response was defined as a complete disappearance of the enlarged lymph nodes. A partial response was defined as a 50% or more decrease of the sum of product of perpendicular diameters of all measurable nodes on imaging. No response (NR) was defined as stable or progressive disease. Complete composite response was considered when both complete primary and nodal response were achieved.

### Follow-up

According to the follow-up policy of our clinic, patients were examined weekly during radiochemotherapy to assess treatment-induced toxicity. After the completion of treatment, all patients were followed up every month over the first year, every other month in the second year, and at 3- to 6-month intervals thereafter. A physical examination and fiberoptic endoscopy were performed during each follow-up examination. Baseline CT and/or MRI of the neck were done every 6 months over the first 2 years. Additional investigations were performed whenever necessary. Biopsy was performed if there was suspicion of residual or recurrent disease.

### Treatment toxicity assessment

Acute reactions induced by radiotherapy were assessed according to the Acute Radiation Morbidity Scoring Criteria of the Radiation Therapy Oncology Group (RTOG) [27] and scored on a weekly basis during the course of treatment and monthly during the first 3 months after the end of treatment. Chemotherapy-related toxicities were assessed according to the World Health Organization (WHO) criteria [28] and were also recorded on a weekly basis during radiochemotherapy. Late radiotherapy-related toxicities were evaluated according to the scales of the European Organization for Research and Treatment of Cancer/Radiation Therapy Oncology Group (EORTC/RTOG) [27] and were recorded starting at 6 months after treatment completion.

### Statistical analysis

The end points examined were local relapse-free survival (LRFS), regional relapse-free survival (RRFS), locoregional relapse-free survival (LRRFS), distant metastases-free survival (DMFS), disease-free survival (DFS), and OS. LRFS was calculated from the first day of treatment until the day when a recurrence at the primary site was first reported, or until the day of the last follow-up. Patients who did not achieve complete primary response were assigned a LRFS of 0 months. RRFS was calculated from the first day of treatment until the day of first occurrence of nodal recurrence, or until the day of the last follow-up. Patients without complete nodal response were assigned a RRFS of 0 months. LRRFS was also evaluated and calculated from the first day of treatment until the day of first occurrence of primary and/or neck relapse, or until the day of the last follow-up. Patients who did not achieve complete composite response were assigned a LRRFS of 0 months. DMFS was measured from the start of treatment to the date of occurrence of clinically detected DM or to the date of the last follow-up. DFS was calculated from the first day of treatment to the date when a relapse was first recorded or, in the case of persistent disease, to the date of first follow-up. The end point of DFS was the occurrence of local, regional or distant relapse. Patients without evidence of disease were censored at the date of last follow-up. OS was calculated from the start of treatment until death, or to the most recent follow-up date. The endpoint for OS was death from all causes. LRFS, RRFS, LRFS, DMFS, DFS and OS curves were calculated using the Kaplan-Meier method [29].

## Results

### Patients characteristics

The patient characteristics are described in Table 1. The median age was 52 years (range, 29-70), with a male predominance (80.5%). The most common primary subsite was pyriform sinus, present in 78.0% of patients. Locally advanced primary tumour (T4) was recognized in 25 patients (61.0%). More than one-half of the patients were diagnosed with metastatic neck nodes. Clinically negative neck (N0) was present in 17 patients (41.5%). In the whole number of patients more than two-thirds were seen with stage IV disease (73.2%). Moderate histological differentiation was present in 17 patients (41.5%). The most frequent symptom at diagnosis was painful swallowing present in 56.1% of patients.

**Table 1:** Patients characteristics (n = 41).

Characteristics	No. of patients	%
Gender		
Male	33	80.5
Female	8	19.5
Age, years		
Median	52	
Range	29-70	
Performance status (ECOG)		
0	26	63.4
1	15	36.6
Subsite		
Pyriform sinus	32	78.0
Posterior pharyngeal wall	5	12.2
Postcricoid area	4	9.8
T stage		
T3	16	39.0
T4	25	61.0
N stage		
N0	17	41.5
N1	6	14.6
N2	12	29.3
N3	6	14.6
Stage		
III	11	26.8
IV	30	73.2
Histological differentiation		
Good	6	14.6
Moderate	17	41.5
Poor	16	39.0
Unknown	2	4.9
Symptoms at diagnosis		
A sore throat	3	7.3
Ear pain	2	4.9
Lump in the neck	8	19.5
Painful swallowing	23	56.1
Change in voice	5	12.2

ECOG, Eastern Cooperative Oncology Group.

### Compliance of treatment

All treated patients received the full planned dose of radiotherapy (70 Gy). In 36 patients (87.8%), the overall treatment time (OTT) for radiotherapy completion was ≤ 7 weeks. Photon-electron treatment was realized in 13 patients (31.7%). The rest 28 patients (68.3%) were irradiated using the technique with oblique photon fields. Twenty-two patients completed all seven cycles of concurrent chemotherapy. Six cycles of cisplatin was given in 16 patients, while 3 patients had less than six cycles of cisplatin with patients' refusal being the only cause for concurrent chemotherapy cessation. The mean total dose of cisplatin given was 192 mg/m^2 ^± 23.2 SD.

### Response to treatment

A complete response at the primary site occurred in 28 patients (68.3%). In patients with positive neck the achieved complete response rate was 36.6%. A complete composite response was present in 27 patients (65.9%). A partial composite response was registered in 14 patients (34.1%). Of those, only one patient had a complete response of the primary tumor and a partial response of the nodal disease. In all other patients, there was a partial response at the primary site and at the neck region.

### Patterns of failure

Median patient follow-up at the commencement of the analysis was 13 months (range 7-36). Local recurrence was developed in 3 patients, 1 patient developed regional recurrence, and 4 patients developed both. Distant metastases were the predominant initial failure occurred in 7 patients and accounting for 46.7% of the cases who manifested a relapse of the disease. Distant metastases were also developed in 3 patients who had not achieved complete composite response following treatment. Hence, the overall incidence of distant metastases was 24.4% (10/41). Not one patient had an identification of distant metastatic disease preceded by the occurrence of local and/or regional recurrence. The most frequent site of distant metastases (80.0%) was the lungs. The median time to development of local recurrence was 12 months (range 4-19). Regional recurrence developed as a single event occurred at 7 months after beginning of treatment. The median time to occurrence of locoregional recurrence and distant metastases was 10.5 months (range 9-19) and 9.5 months (range 4-21), respectively.

### Survival

At the time of analysis, 19 patients were alive. Among those, 1 patient had recurrence in the neck nodes, 1 patient had recurrence of the primary tumour, 2 patients had recurrence of the primary and of the nodal disease, 4 patients had distant metastases, and 11 patients were alive free of disease. During the follow-up period, due to tumor progression, a tracheotomy had been performed in 8 patients, 3 patients required a placement of a feeding tube, and in other 3 patients a gastrostomy had been performed. Death was disease-related in vast majority of patients (21/22). Three patients had died of local or locoregional recurrence, 1 patient had unknown cause of death, 12 patients had died of the progression of their persistent disease, 3 patients had died of distant metastases, and 3 patients had died due to both persistent disease and distant metastases.

**Figure 1:**
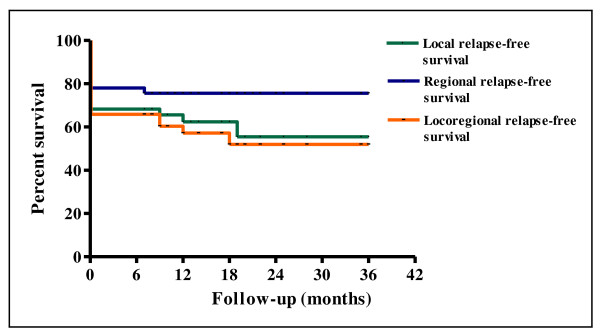
**Kaplan-Meier curves of local relapse-free survival, regional relapse-free survival and locoregional relapse-free survival**.

**Figure 2:**
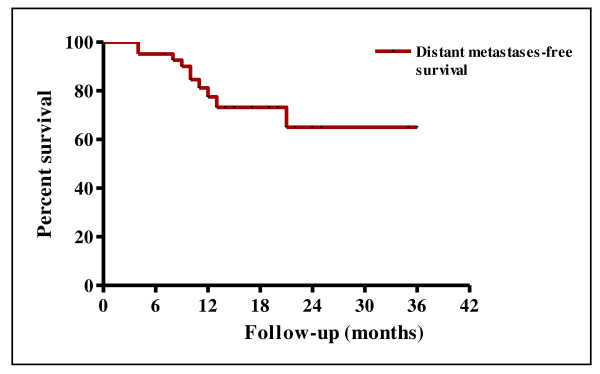
**Kaplan-Meier curve of distant metastases-free survival**.

**Figure 3:**
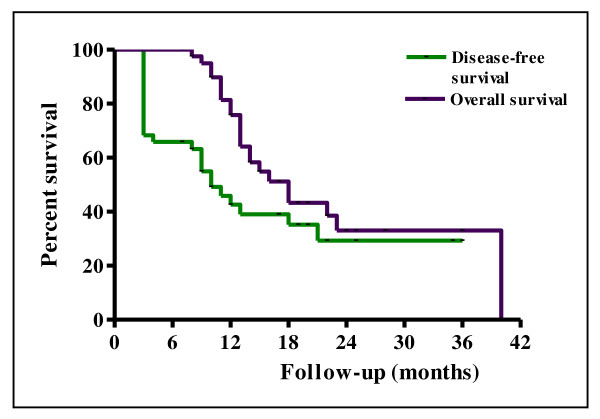
**Kaplan-Meier curves of disease-free survival and overall survival**.

The 2-year LRFS and RRFS survival rates were 55.2% and 75.8%, respectively (Figure 1). The median duration of LRFS was 10 months (range 0-36) and the median duration of RRFS was 12 months (range 0-36). The LRRFS at 2 years was 51.3% (Figure 1). The median duration of LRRFS was 10 months (range 0-36). The DMFS at 2 years was 64.7% (Figure 2). The median duration of DMFS was 12 months (range 4-36). The 2-year DFS and OS survival rates were 29.3% and 32.8%, respectively (Figure 3). The median duration of DFS was 9 months (range 3-36) and the median duration of OS was 14 months (range 7-36).

### Toxicity

The acute toxic effects of treatment are illustrated in Table 2. Grade 2 skin reaction (deep hyperpigmentation and dry desquamation) was developed in 75.6% of patients. Grade 3 skin reaction (moist desquamation) was manifested in only 4.9% of patients. Grade 2 mucous membrane reaction (patchy mucositis) was developed in 48.8% of patients. Similar proportion (46.3%) of patients developed confluent mucositis (grade 3 mucous membrane reaction). The most common grade of acute reaction in the pharynx was grade 2, experienced in 73.2% of patients. The most frequent haematological toxicity was leucopenia grade 1, present in 70.7% of patients. The median weight loss at the end of radiochemotherapy was 12.0% (range 5-21). Late normal tissue reactions are listed in Table 3. The worst grade of late toxicity (grade 2) was most commonly found in the skin (34.1% of patients) and in the subcutaneous tissue (65.9% of patients).

**Table 2:** Acute toxicity induced by concurrent chemoradiotherapy.

	Grade of reaction (% of 41 patients)			
	
Toxicity	0	1	2	3
Acute normal tissue reactions according to RTOG criteria				
				
Organ/Tissue				
Skin	0	19.5	75.6	4.9
Mucous membrane	0	4.9	48.8	46.3
Salivary gland	0	34.1	61.0	4.9
Pharynx	0	21.9	73.2	4.9
Larynx	24.4	36.6	39.0	0
				
Acute toxicity according to WHO criteria				
				
Nonhematological				
Nausea	46.3	51.2	2.4	0
Vomiting	63.4	34.1	2.4	0
Weight loss	0	43.9	53.6	2.4
				
Hematological				
Leucopenia	19.5	70.7	9.8	0
Anemia	36.6	56.1	7.3	0
Thrombocytopenia	51.2	48.8	0	0

RTOG, Radiation Therapy Oncology Group; WHO, World Health Organization.

* Because of rounding, not all percentages total 100.

**Table 3:** Late normal tissue reactions.

	Grade of reaction (% of 41 patients)		
	
Organ/Tissue	0	1	2
Skin	2.4	63.4	34.1
Subcutaneous tissue	0	34.1	65.9
Mucous membrane	0	87.8	12.2
Salivary gland	9.8	75.6	14.6

* Because of rounding, not all percentages total 100.

## Discussion

Hypopharyngeal cancer is a head and neck neoplasm with one of the most unfavourable prognosis. Although there is no level one evidence on best treatment for advanced hypopharyngeal cancer [16], considering the fact that vast majority of patients presenting with advanced stage disease are inoperable, unfit for operation, or refuse surgery, we found the use of chemotherapy and radiotherapy administered concomitantly as a rational treatment option for this patient category.

The achieved rate of complete composite response in our study was 65.7%. This result is comparable with the results observed in other studies investigating the effectiveness of combined radiotherapy and chemotherapy for advanced hypopharyngeal carcinoma. Yoon et al. [30] reported a complete response rate of 82.0% in the group of 28 patients treated with induction chemotherapy followed by CRCT. In the retrospective study comparing the results of treatment of locally advanced hypopharyngeal cancer with two different protocols, Hung et al. [4] found the response rate of 77.0% in 38 patients treated with definitive CRCT followed by adjuvant chemotherapy.

The results of our study demonstrated that grade 3 acute response of the mucous membrane was present in almost one half of the patients (48.8%). This corresponds with the findings of other authors [4,31]. The rate of grade 2 weight loss registered in our study was 53.6%, but we did not observe increased rates of chemotherapy-related haematological toxic effects. Although the incidence of development of confluent mucositis was expected to be increased with CRCT and was thought to affect treatment course, there was no treatment interruption occurring in the event of swallowing disturbance in our study. Also, none of the patients had to discontinue treatment as a consequence of haematological toxicity. Using three dimensional conformal radiotherapy and concomitant weekly cisplatin we succeeded to deliver the prescribed dose of 70 Gy in all the patients, and there were only 3 patients receiving less than six cycles of concurrent chemotherapy.

In our study locoregional recurrence occurred in 8 patients. Distant metastases as an initial failure occurred in 7 patients. The overall incidence of distant metastases was 24.4%. Similar findings were obtained by other authors. In the study of Hung et al. [4], distant metastases also occurred in 24.0% of patients treated with CRCT. In the retrospective analysis of Elias et al. [32] on treatment results of carcinoma of the pyriform sinus, 32.0% of patient population developed distant metastases. The frequency of DM of 46.7% as first site of failure in our study was ten fold higher compared to the results reported by Johansen et al. [2]. Although it is generally considered that locoregional control of head and neck cancer represents an important factor for development of distant metastases, we did not observe any distant metastases development preceded by occurrence of locoregional recurrence. This data led us to assume that there had been a high probability for the presence of occult distant metastases at the time of initial patients screening.

The LRRFS at 2 years revealed in our study was 51.3%, a result that differs from the 2-year locoregional progression-free survival rate for patients with hypopharyngeal cancer of 73.0% in the study of Lee et al. [31]. In the study of Yoon et al. [30], the 3-year locoregional control for the group treated with CRCT was 52.0%. Johansen et al. [2] in their study of treatment results in 138 patients with hypopharyngeal carcinoma reported low rates of locoregional control at 5 years (25.0%). The 2-year DMFS rate in our study was 64.7%. In contrast, Lee et al. [31] reported rate of 2-year freedom from distant metastasis of 92.0%. In the study of Kim et al. [33] on treatment results in advanced hypopharyngeal carcinoma according to treatment modalities, the 5-year DMFS rate in the group treated with induction chemotherapy and radiotherapy was 82.4%. The rate of 2-year DFS in our study is similar to the 3-year DFS rate of 28.8% for stage IV hypoharyngeal cancer in the study of Gupta et al. [34]. In the study of Hung et al. [4], there was also a very low rate of 3-year DFS (21.0%). The OS rate at 2 years in our study was 32.8% and was lower than the reported 2-year OS rate of 53.0% in the study of Lee et al. [31]. A higher rate of OS was also reported by Yoon et al. [30] and by Hung et al. [4] (3-year OS rate of 54.0% and 43.0%, respectively).

Considering the rates of LRRFS, DMFS, DFS, and OS in our study, and comparing them with the rates observed in other clinical studies, we must conclude that irrespectively to the combined treatment modality used (CRCT, induction chemotherapy with radiotherapy, or CRCT followed by adjuvant chemotherapy), the results were far from being satisfactory. At this point, the necessity of locoregional control improvement and decrease of distant metastases as critical factors for increase in DFS and OS survival must be taken in consideration. The implementation of intensity-modulated radiotherapy (IMRT) as sophisticated radiotherapy technique combined with concomitant chemotherapy, the use of altered fractionation regimens, the use of intensified concurrent radiochemotherapy, the adoption of sequential therapy, and the administration of targeted therapy concurrently with conventional or altered fractionation, could be considered as combinations of treatment factors able to contribute to the ultimate outcome of patients with advanced hypoharyngeal cancer.

The possibility for higher dose delivery in the tumor while sparing critical structures by using IMRT are reasonably expected to enable improvement in locoregional control in advanced hypopharyngeal cancer.

The improvement of locoregional control in advanced head and neck cancer has been achieved by the use of altered fractionated radiotherapy [35,38]. In the meta-analysis of 15 trials examining the role of altered fractionation radiotherapy in head and neck cancer, Bourhis et al. [39] reported that altered fractionation improves 5-year local control by 6.4% and 5-year OS by 3.4% compared with conventionally fractionated radiotherapy with significantly higher benefit seen with hyperfractionated radiotherapy. The comparison of conventionally fractionated radiotherapy with altered fractionation radiotherapy in the German meta-analysis of 32 randomized trials on patients with squamous cell carcinoma of the head and neck (oral cavity, oropharynx, hypopharynx, and larynx) also showed that hyperfractionation led to a significant improvement of OS if radiotherapy was used as single treatment modality [40].

Regarding the confirmed role of CRCT as a recommendable treatment option in advanced head and neck cancer [41,42] and considering the confirmed advantage of altered fractionation over conventionally fractionated radiotherapy, the intensified CRCT (altered fractionation and concurrent chemotherapy) should be also considered as another attempt to further improve both locoregional control and OS. In the prospective randomized clinical study in advanced oropharyngeal, oral cavity and hypopharyngeal cancer conducted by Budach et al. [43], altered fractionation radiotherapy with CRCT was found superior to altered fractionation radiotherapy alone. The analysis on altered fractionation radiotherapy in combination with concurrent chemotherapy in the German meta-analysis resulted in an OS benefit of 12.0% compared to altered fractionation radiotherapy alone, that was similar to the survival benefit achieved with conventionally fractionated radiotherapy with concurrent chemotherapy, compared with conventionally fractionated radiotherapy alone [40]. These results suggested that the use of concurrent chemotherapy enabled a significant improvement in OS, regardless of the fractionation regimen employed.

The development of distant metastases during follow-up period represents a serious problem reflecting negatively on DFS and OS in patients with advanced hypopharyngeal cancer. The adoption of CRCT as a standard of care for patients with locally advanced HNSCC is associated with improved locoregional control and OS compared to radiotherapy alone [39,40], and its efficacy to decrease the rate of distant metastatic disease has been also confirmed in phase III trials that have tested this treatment option [23,44,45].

Targeted therapy in head and neck cancer is closely connected to the use of cetuximab, an anti-Epidermal Growth Factor Receptor (EGFR) antibody therapy, confirmed to be effective in combination with radiotherapy in previously untreated patients in a single controlled clinical trial [46,47]. The recently started phase III trial (RTOG 0522) exploring cisplatin-based CRCT with or without cetuximab is expected to clarify the role of cetuximab in integrated radiochemotherapy association [48].

## Conclusions

Taking into account the unsatisfactory results achieved using CRCT in our study, we strongly advocate an emerged change of therapeutic approach in patients with advanced hypopharyngeal cancer.

Concerning the radiotherapy technique in the treatment of hypopharyngeal cancer, we recommend the adoption of IMRT in the routine clinical practice because of its more conformal dose distribution with steep gradients between PTV and critical structures.

We also consider that hyperfractionation, in the presence of sufficient radiotherapy resources, could be implemented as treatment modality with beneficial impact on OS in patients with advanced hypopharyngeal cancer.

Accepting platinum-based CRCT, in the absence of conclusive arguments supporting exact schedule of cisplatin administration, we advocate the use of weekly cisplatin considering it as the simplest solution that provides radiosensitizing effect to a larger proportion of the radiotherapy dose and leads to less chemotherapy induced toxicity.

## Competing interests

The authors declare that they have no competing interests.

## Authors' contributions

VK and IS have made substantial contributions to design of the study and collected the data. VK performed much of the work and drafted the manuscript. DL has been involved in drafting the manuscript in the section of radiotherapy techniques. VK and IS interpreted the data. VK performed the statistical analysis. All authors read and approved the final manuscript.
